# Consumer intention to purchase GM soybean oil in China: effects of information consistency and source credibility

**DOI:** 10.1080/21645698.2021.2002627

**Published:** 2022-01-03

**Authors:** Mingyang Zhang, Zihao Chen, Yubing Fan, Zhiqiang Cheng, Ting Lv, Yuling Chen

**Affiliations:** a School of Business, Development Institute of Jiangbei New Area, Nanjing University of Information Science and Technology, Nanjing, China; b College of Economics & Management, Nanjing Agricultural University, Nanjing, China; c State key Lab of Grassland Agro-Ecosystems, College of Pastoral Agriculture Science and Technology, Lanzhou University, Lanzhou, Gansu, China; d Jiangsu Provincial Station of Rural Cooperative Economy Management, Department of Agriculture and Rural Affairs of Jiangsu Province, Nanjing, China

**Keywords:** GM foods, source credibility, attitude change, purchase intention, risks and benefits

## Abstract

Consumers’ potential reactions toward genetically modified (GM) foods affect their commercial feasibility and determine the decisions of economic agents. Inconsistent information on GM foods has created a sense of uncertainty in Chinese consumers’ mind. This paper studies how the information about risks and benefits of GM foods from major sources influences Chinese consumer intention to purchase GM soybean oil. This analysis uses data from a survey of 880 residents randomly sampled from 13 cities in Jiangsu province. Using a multinomial logit model, we analyze the effects of information consistency and source credibility. The results show because of new information about 17.36% of consumers increase their intention to purchase GM soybean oil, and 15.10% of consumers decrease purchase intention. Compared to consistent information, inconsistent information can maximize change of purchase intention. The attitude change is greatest when there is a moderate difference between the new information and the initial consumer attitude. Furthermore, trust in biotechnology research institutes, government departments about GM, and GM experts are easier to promote consumers to change their intention to purchase GM soybean oil in a positive direction. Finally, we discuss implications for agencies as to strengthening the regulation and supervision of information sources, and including public-involved policies.

**Abbreviations:** GM, Genetically modified; GMOs, Genetically modified organisms; AGGMO, Center of Agriculture’s Genetically Modified Organisms’ safety management and policy research organization at Nanjing Agricultural University; MARA, Ministry of Agriculture and Rural Affairs; ¥1 (RMB)≈$6.8 (USD).

## 1. Introduction

Although gene technology has experienced a rapid development in varietal breeding, industrial application and food industry, genetically modified (GM) foods are facing much controversy. In 2019, 29 countries planted more than 10 kinds of transgenic crops, including soybeans, corn, rapeseed and some other crops. The Ministry of Agriculture and Rural Affairs (MARA) of the P. R. China granted 21 bio-safety certificates for staple crops from 2009 to 2020, including GM rice, GM soybean, and GM maize.^1,2^Nevertheless, they have not been allowed to plant^3^ mainly because of consumer concerns about the potential risks of GM foods on people’s health.^4^ To make it even worse, the news media have been largely focused on reporting the controversy of GM foods. Various inconsistent sources has left a sense of uncertainty in consumers’ mind.^5^ An open question concerning researchers is whether systematic changes of consumer intention to purchase GM food occur in response to information dissemination about risks and benefits of GM foods.

To contribute to this literature, focusing on consumer intention to purchase GM soybean oil, the empirical evidence will provide scientific insights by investigating how Chinese consumers purchase intention of GM soybean oil response to information about risks and benefits of GM foods (that we provided), and estimating overall effect of information consistency and source credibility on consumer intention to purchase GM soybean oil. Because food security is constrained by arable land, China has imported a variety of GM crops, including GM soybean GM corn, GM rapeseed, GM beet, etc., from the US, Argentina, and Brazil every year. While soybeans imports accounted for over 90%, with about 96 million tons soybeans in 2019.^6^ Those soybeans are mainly used to produce GM soybean oil. As more and more consumers are informed the use of these soybeans, their intention to purchase GM soybean oil may be change.

In the next section, we briefly discuss the literature to which we aim to contribute (Section 2). We then describe material and methods (Section 3), before we come to our results and discussion (Section 4) and conclusions (Section 5).

## 2. Literature Review

### 2.1. An Integrated Theoretical Foundation

As an important resource, information has economic values and aids consumers in decision making. Information can change an individual’s prior probability and help him/her make a better decision.^7^ Hovland’s Theories of Attitude Change point out that the initial attitudes are not immutable and four components work jointly in the process of attitude change^8^: (1) the persuader, i.e., the information source, (2) the persuasion information, (3) the persuasion situation, i.e., the individual’s “enhanced” access to information, and (4) the persuasion objects, including individual and family characteristics. Among all the factors, the persuader is most critical. As a filter, credibility determines the individual’s access to the information sources.^9^ With limited knowledge of GM foods, a consumer may have to rely on the source credibility to make judgments on information before making a purchase decision.

Furthermore, Cognitive Response Theory indicates that prior knowledge has been identified as a factor affecting an individual’s ability to process information. This is well confirmed by Elaboration Likelihood Model. The model shows external information leads to a series of individual thinking, which not only includes understanding and judgment of the information, but also compares it to the original memory based on the initial information. When the received persuasion information is inconsistent with the initial attitude, the individual becomes nervous, which triggers an inconsistency mechanism. When the difference between the persuasion information and the initial attitude of the persuasion object is moderate, information can maximize the attitude change.^11^ Take GM foods as an example, whether a consumer changes purchase intention depends on the degree of the individual’s refutation to the new information. If the refutation is disturbed, the information has a persuasive effect. As a result, the external information may change the consumer’s purchase intention. Otherwise, the consumer retains the initial attitude through various means, such as belittling the information source, and deliberately distorting the information.

Source credibility, as an experimental factor, often changes in the process of attitude change.^14^ Then how does the credibility of various sources affect information credibility? Some theories indicate: firstly, an individual or organization with certain expertise is often considered as a persuader of high credibility, and the information released by the persuader is more likely to be trusted by the public. Consequently, it affects their attitudes ^16^. Secondly, if a source combats the vested interest of a person or group, it is usually considered a trusted source.^15^ Thirdly, when the persuaders benefit from the ideas they advocate, especially if the ideas are perceived to be deliberately biased in a particular direction, their credibility is more likely to be questioned by the consumers. Even if the persuader’s views are objective, people will not believe the information. Fourthly, when individuals realize that the persuader intends to influence them, they have psychological resistance, as a result, they may show resistance toward the information. On the contrary, if the persuader does not intend to influence them, the public will believe and accept the persuasion information.^8^

### 2.2. Consumer Attitudes Toward GM Foods

A number of researchers have examined consumers’ attitudes toward GM foods.^16,17^ The consumer attitudes are impacted by many influential factors including risk perceptions,^18^ benefit perceptions,^19,20^ consumers’ knowledge,^21,22^ trust^23–28^ social responsibility,^29^ individual characteristics,^30,31^ and socioeconomic factors.^19,32^In particular, consumer attitude can be influenced by information dissemination regarding GM foods including crops, processing aids, and relevant public policies.^16^ A few studies have investigated the effects of communication about GM foods on consumer attitudes using controlled attitude change experiments. Interestingly, these studies did not find consistent information effect on attitude change.

Studies have examined the effects of power of persuasion and information type. For instance, regarding the traditional risk-benefit information, no consistent outcome was found on the attitude change toward GM foods whether the powers of persuasion were the same^33^ or different.^34^They also investigated the effects of three different types of communication materials about transgenesis on food production, including argumentatively balanced information, benefits, and conventional product advertising. No systematic attitude changes occurred in response to the communication whether the information sources are the same^35^ or different.^36^ Dean and Shepherd^37^exposed their participants to message pairs with different sources either in consensus or in conflicts regarding perceptions of risks or benefits associated with GM foods. They found that the attitude change in terms of a decrease in perceived risk was observed only in response to the message pair in consensus, while the attitude change in terms of an increase in perceived benefit was observed in response to the message pairs both in conflict and in consensus.

New forms of risk assessment and management have been documented in recent literature. By employing new detection methods and providing information about improved traceability of GM food ingredients through the food chain, Miles, et al.^38^observed no effect on attitude change. Wuepper, et al.^39^conducted a labeled choice experiment with different breads, one of which contained “functional” GM wheat with added benefits such as a prolonged shelf-life, added micro-nutrients and more fiber. They found that the average effect of providing balanced information was negligible, and that the initially less opposed effect became slightly more opposed. However, Qin and Brown^40^obtained a small but significant attitude change in response to information materials which described a specific application in GM salmon and outlined the locations of major stakeholder groups of the application. However, it is uncertain whether the information materials influenced consumers’ attitude toward general GM foods, since the attitude measure in their study only referred to the specific application.

Empirical studies have analyzed the relationship between consumer attitude and source credibility. Dean and Shepherd^37^showed no mediating effects of source credibility on information exposure and attitude change. The results were consistent with Frewer et al.,^33^ and Frewer et al.^36^ However, some others pointed out that source credibility has a significant effect, and highly credible source is more likely to influence consumer attitude toward GM foods.^41,42^ Information from professionals and experts affects public comprehension of scientific information and shows a positive effect on attitude change.^43^ In general, consumers are likely to believe the information sources that seem to hold similar values with them.

In conclusion, for both the traditional risk-benefit communication including balanced information, benefits and conventional product advertising, and information materials about specific GM foods or specific transgenesis application, most of the studies did not manage to find consensus results that whether systematic attitude changes occurred in response to the communication. Few studies have investigated the effect of information consistency on consumer attitudes change. The similar study is conducted by Priester et al.,^12^ who thought external information led to individual comparing it with the original memory based on the initial information.

## 3. Material and Methods

### 3.1. Research Design

China’s MARA oversees the national GMO safety management. This study is based on the monitoring and management of agricultural GMO applications by the Center of Agriculture’s Genetically Modified Organisms’ Safety Management and Policy Research Organization at Nanjing Agricultural University (AGGMO) from 2010 to 2017. There are mainly six information sources, including biotechnology research institutes (i.e., Biotechnology Research Institute at the Chinese Academy of Agricultural Sciences, Jiangsu Academy of Agricultural Sciences), government departments devoted to GMO management (i.e., MARA GMO Safety Management Office, Jiangsu GMO Safety Management Office, etc.), GM technologists, environmental organizations (Greenpeace), Non-GM personages, and anonymous internet users.

According to a long-term statistical investigation of AGGMO and the current literature, there are two attributes (reliability and expertise) significantly affect the quality of the information provided. Reliability refers to the validity of the persuader’s assertion perceived by the public, and expertise refers to the extent to which the public perceives the persuader to make a right statement.^47^ Therefore, the information sources can be grouped into three following categories.

(1) Professional and reliable sources, named by GM department and experts, including biotechnology research institutes and government departments relating to GM,^37,48^ and GM technologists.^49^ Since these are very professional entities, their information is highly credible.

(2) nonprofessional and reliable sources, named by environmental organizations (e.g., Greenpeace).^41^ They are often regarded as highly credible sources because they try challenging the vested interests of some multinational biotechnology companies (e.g., Monsanto).

(3) nonprofessional and unreliable sources, named by Non-GM individuals including non-GM individuals and anonymous internet users. Non-GM individuals are inexperienced with transgenic technology.^5^ Anonymous internet users are often seen as a deliberate influence on consumers, so their reliability is likely to be questioned.^44^

The information from the three sources may show varying effects. To investigate the information materials and their effects, we designed three versions of survey cards as shown inFig. 1. Cards A, B, and C correspond with the three information sources mentioned above. Card A is about transgenesis benefits from biotechnology research institutes, government departments relate to GM and GM technologists. Card B is about transgenesis risks from environmental organizations. And Card C is about transgenesis risks from non-GM experts and anonymous internet users.

**Figure 1.**
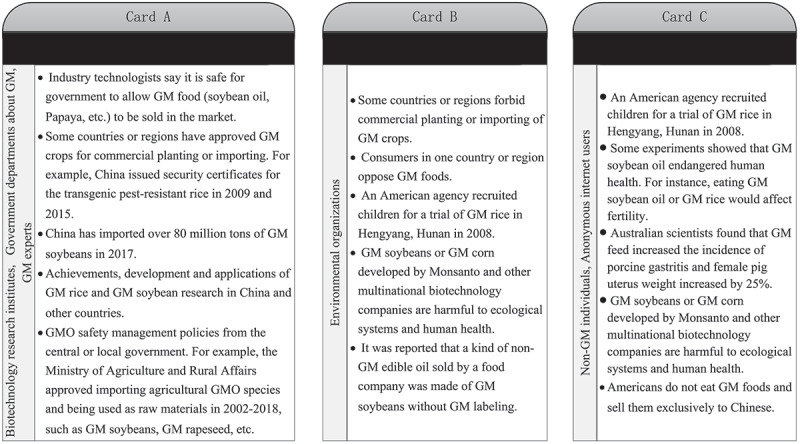
Cards showing possible sources and relevant information.

Cards showing possible sources and relevant information.

### 3.2. Survey and Sampling

The survey includes questions regarding consumer intention to purchase GM soybean oil, information materials, and socio-demographic inquiries. The survey was conducted in Jiangsu, which is an eastern coastal province in China. The social and economic development of Jiangsu is markedly progressive from south to north, which is similar to China’s social and economic development from east to west. Compared to other provinces, population density and proportions of city residents are relatively high in Jiangsu. More importantly, the city residents are more familiar with GM foods.^5^ The survey was conducted at large-scale shopping malls and supermarkets selected from 13 cities in Jiangsu province. To minimize the sample selection bias, two-three sites were selected in each city, and the interview was conducted on both workdays and weekend to include samples representing different social classes.^50,51^

Selected undergraduates from Nanjing Agricultural University administered the survey in 2018. The investigators were divided into three groups, and each presented the respondents with information on Cards A, B, and C. In the survey, we tried to avoid the problem of endogenous knowledge, e.g., due to different education levels, or different interest degrees. Because interactions between endogenous and exogenous variables remain exogenous.^39^ Firstly, the respondents were asked about their initial purchase behavior on GM soybean oil, and their trusts in the given three types of information sources. Secondly, the respondents were only provided one of the three cards. Take Card A as an example, the information combination was expressed as “this information was released by biotechnology research institutes, government departments relating to GM, or GM experts.” Subsequently, the respondents were asked about their judgments on the provided information and their intentions to purchase GM soybean oil given the information.

**Figure 2.**
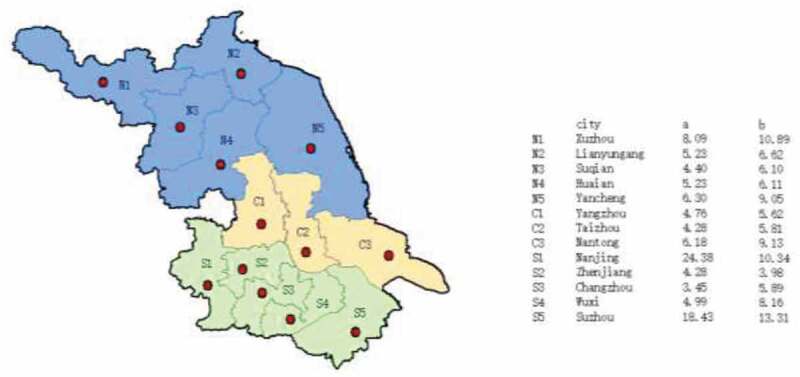
Geographical distribution of the sample (%).

Notes: City means the prefecture-level cities in Jiangsu province. a/b: a indicates the proportion of sampled city residents in each city accounting for the sample. b indicates the proportion of a city’s permanent population accounting for Jiangsu permanent population. Based on data of “Major data of all previous provincial population census” from Jiangsu Statistical Yearbook 2016. The permanent population includes city residents, county-seat residents, town residents, and rural people.

To participate in the survey, the participants were confirmed at least 16 years old and a citizen in a city. A self-administered survey was conducted using a random sampling approach. Each respondent was offered a ¥20 gift after completing the survey. Totally 880 respondents with 50% provided information about benefits of GM foods were surveyed, while the proportions of sample of three versions (A, B, and C) are 50%, 25%, and 25%, respectively. After accounting for missing observations, this study used data of 841 valid observations (valid return rate is 95.6%). The geographical distribution of the sample is shown in Fig. 2. Samples in each region roughly represent the regional populations. Nanjing and Suzhou are two most populated regions, with samples accounting for 24.38% and 18.43%, respectively.

Fig. 3

**Figure 3.**
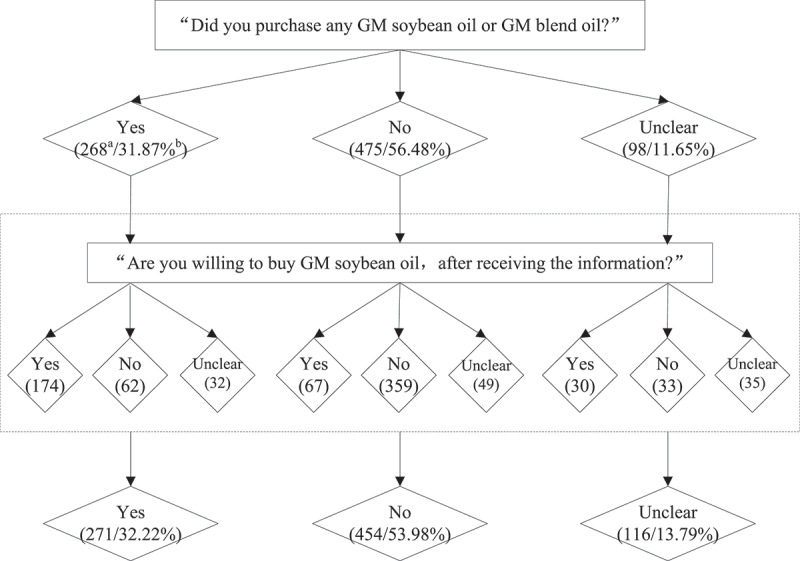
Change in purchase intention on GM soybean oil.

a/b: a indicates the number of respondents. b indicates the corresponding proportion.

### 3.3. Method

Following the studies about consumer behavior,^52,53^ a multinomial logit is used to investigate effects of the information on purchase intention change. Based on random-utility theory, the utility of choosing the *j-th* choice by the *i-th* individual is^54^:

(1) $$U_{ij}=x{{\;}^{\prime }}_{i}\beta_{j}+\varepsilon_{ij}\;i=1,\ldots ,n;j=1,..,J$$

Here $x_{i}$ is the matrix of the characteristics of the individual *i*, and $\beta_{i}$ is the parameter vector for each alternative. Following the development of random utility theory, the probability that an individual *i* selects alternative *j* equals the probability that *U_ij_* is greater than the utilities *U_ik_* of all other alternatives in the individual’s choice set, C.

(2) $$P_{i}\left(Y=j\right)=P\left(U_{ij}\geq U_{ik}\forall k\in J\;k\neq j\right)$$

It is assumed that the random components of the utility ($\varepsilon_{ij}$) are independent and identically distributed with a Gumbel distribution. Therefore, in multinomial logit modes, the probability of an individual *i* choosing an alternative j can be expressed by^53^

(3) $$P_{i}\left(Y=j|x\right)=\left\{\begin{array}{c} \frac{\exp\left({x^{\prime }}_{i}\beta_{j}\right)}{1+\sum_{j=2}^{J}\exp\left({x^{\prime }}_{i}\beta_{j}\right)}\;\;\;\left(j=2..J\right)\\ \frac{1}{1+\sum_{j=2}^{J}\exp\left({x^{\prime }}_{i}\beta_{j}\right)}\;\;\;\left(j=1\right) \end{array}\right.$$

Here the choice of “j=1” is the base category. The logarithmic form of likelihood function of *i-th* individual is^55^:

(4) $$\ln L_{i}\left(\beta_{1},\ldots ,\beta_{J}\right)=\sum_{j=1}^{J}1_{i}\left(Y=j\right)\cdot \ln P_{i}\left(Y=j|x\right)$$

where *1(*⋅) is an indicator function, and the value is equal to 1 if the expression in parentheses is true. The log-odds ratio of conditional probability of “$y_{i}=j$” happening is:

(5) $$\ln\left[\frac{P_{i}\left(Y=j\right)}{P_{i}\left(Y=1\right)}\right]=x_{i}^{\prime }\beta_{j}$$

Here the parameter estimates for the $\beta_{j}$ vectors can be obtained using the Newton method.^56^ Marginal effects of independent variables can be calculated from equation (5) below:

(6) $$\frac{\partial P_{i}\left(Y=j\right)}{\partial x_{i}}=P_{i}\left(Y=j\right)\left[\beta_{j}-\sum_{j=1}^{J}P_{i}\left(Y=j\right)\beta_{j}\right]for\;j=1,2,..,J$$

### 3.4. An Empirical Model

Suppose that the respondent’s intention to purchase GM soybean oil has three changes of direction (*y = 1, 2, …, J*) after receiving the information (no change = 1; positive change = 2; negative change = 3). In this study, the empirical model can be expressed as follows:

(7) $$\ln\left[\frac{P_{i}\left(Y=j\right)}{P_{i}\left(Y=1\right)}\right]=\sum_{k}ln\beta_{k}Consistency_{ik}+\sum_{n}ln\delta_{n}Source_{in}+\alpha_{i}Credibility_{i}+\sum_{n}\gamma_{n}Interaction_{in}+f\left(X_{im};\beta_{m}\right)+\mu_{i}$$

Here, the explanatory variables include information consistency (*Consistency_k_*) and source credibility (*Source_n_*) and the interaction of source and source credibility. Information consistency is measured by five dummy variables: *completely inconsistent, inconsistent, neutral or unclear, consistent and completely consistent*, while the “*consistent*” is taken as the base category. Source credibility is measured by the trust in the sources in the received Card. Interaction is measured by three dummy variables: *Credibility of GM department or experts, Credibility of environmental organizations*, and *Credibility of non-GM individuals* (the base category) (see Table 1).

**Table 1.** Dependent and independent variables with definitions and descriptions

Variable	Description and measurement	Mean	Standard deviation	Min	Max
Dependent variables					
*Attitude change*	Consumers change their intentions to purchase GM soybean oil after receiving the information. (1 = no change (base); 2 = a positive change, changing from “not purchase” to “unclear,” from “unclear” to “willing,” or from the “not purchase” to “willing”; 3 = a negative change, changing from “purchase” to “unwilling,” from “unclear” to “unwilling,” or from “willing” to “unclear”)	0.675 0.174 0.151	0.469 0.379 0.358	1 0 0 0	3 1 1 1
Explanatory variables					
Information consistency	Whether the provided information is consistent with your initial attitude?				
*Completely inconsistent*	(1 = completely inconsistent; 0 = otherwise)	0.046	0.210	0	1
*Inconsistent*	(1 = inconsistent; 0 = otherwise)	0.190	0.393	0	1
*Neutral*	(1 = neutral or unclear; 0 = otherwise)	0.386	0.487	0	1
*Consistent(base)*	(1 = consistent; 0 = otherwise)	0.353	0.478	0	1
*Completely consistent*	(1 = completely consistent; 0 = otherwise)	0.024	0.152	0	1
Source	One respondent was just provided one type of three cards.				
*GM department or experts*	(1 = receiving Card A with information released by biotechnology research institutes, government departments about GM, or GM experts; 0 = otherwise)	0.491	0.500	0	1
*Environmental organizations*	(1 = receiving Card B with information released by environmental organizations; 0 = otherwise)	0.253	0.435	0	1
*Non-GM individuals(base)*	(1 = receiving Card C with information released by non-GM individuals and anonymous internet users; 0 = otherwise)	0.256	0.436	0	1
*Source credibility*	Trust in the sources in the received Card (1 = trust; 0 = distrust)	0.340	0.490	0	1
Interaction					
*Credibility of GM department or experts*	The interaction of *GM department or experts* and *source credibility* (1 = trust in *GM department or experts*; 0 = otherwise)	0.216	0.412	0	1
*Credibility of environmental organizations*	The interaction of *environmental organizations* and *source credibility* (1 = trust in environmental organizations; 0 = otherwise)	0.133	0.340	0	1
*Credibility of non-GM individuals (base)*	The interaction of *non-GM individuals* and *source credibility* (1 = trust in non-GM *individuals*; 0 = otherwise)	0.050	0.218	0	1
Socio demographic characteristics					
*Age*	Age of respondents in years.	30.54	9.157	16	71
*Child*	Whether there is a child under 6 years old at home? (1 = yes; 0 = no)	0.449	0.498	0	1
*Main edible oil*	Soybean oil is the main edible oil in the family. (1 = yes;0 = no)	0.572	0.495	0	1
*Northern region*	Including 5 cities: Huaian, Lianyungang, Suqian, Xuzhou, Yancheng. (1 = yes; 0 = otherwise)	0.293	0.455	0	1
*Central region (base)*	Including 3 cities: Nantong, Taizhou and Yangzhou. (1 = yes; 0 = otherwise)	0.152	0.359	0	1
*Southern region*	Including 5 cities: Nanjing, Suzhou, Wuxi, Changzhou and Zhenjiang (1 = yes; 0 = otherwise)	0.555	0.497	0	1

Other individual characteristics are showed and compared with the population in Table 2.

**Table 2.** Statistic summary of socio-demographic variables and a comparison with the population

Variable	Measurement index	Sample ^a^	Jiangsu population ^b^
Male	1 = male; 0 = female	43.76%	50.40% ^c^
Education			
*Senior high school or below (base)*	1 = senior high school or below; 0 = otherwise	18.67%	36.57%^c^
*Professional college or above*	1 = professional college or above; 0 = otherwise	81.33%	20.7%^c^
Family disposable income	Per capita monthly disposable income ^d^		
*Low-income*	1 = about ¥1300^e^; 0 = otherwise	6.80%	20.00%
*Lower-middle*	1 = about ¥ 2300; 0 = otherwise	9.40%	20.00%
*Middle*	1 = about ¥ 3000; 0 = otherwise	17.60%	20.00%
*Higher-middle (base)*	1 = about ¥ 4000; 0 = otherwise	31.00%	20.00%
*High-income*	1 = about ¥ 7000; 0 = otherwise	35.20%	20.00%

^a^Based on the survey data of city residents in Jiangsu province. ^b^ Based on the population data from Jiangsu Statistical Yearbook 2016.^c^ The population includes city residents, county-seat residents, town residents and rural people. ^d^ Jiangsu urban residents in 2016. The income is divided into five levels: low (¥1332), lower-middle (¥2255), middle (¥3038), higher-middle (¥4054), and high (¥7006).^e^ 6.8 ¥≈1 USD.

Multiple individual characteristics (*X_im_*) are included in the model. Education is a dummy variable and the base category is *senior secondary school or below*. The per capita monthly disposable income is measured by five dummy variables: *low* (about ¥1300), *lower-middle* (about ¥2300), *middle* (about ¥3000), *higher-middle* (about ¥4000), and *high* (about ¥7000), while the *higher-middle* group is used as the base category. *Child* reflects whether there is a child under 6 years old at home. *Main edible oil* reflects whether the soybean oil is the main edible oil for the respondent household. Dummy variables for three regions, that is, northern, centra,l and southern, are included in the model (see Table 2).

## 4. Results and Discussion

### 4.1. Descriptive Statistics

A statistic summary of the sample socio-demographics is presented in Tables 1 and Table 2. Compared with the Jiangsu population in 2015, which included city, county-seat and rural residents, the investigated sample of city residents have lightly more females accounting for 56.24% and more well-educated people with 81.33% having a professional college or higher degree. The sample includes more young people. For per capital monthly disposable income, the proportions of low, lower-middle, middle, higher-middle, and high-income groups are 6.78%, 9.39%, 17.6%, 31.03%, and 35.20%, respectively.

The respondents receiving information from GM department or experts, environmental organizations, and non-GM individuals account for 49.11%, 25.33%, and 25.56%, respectively. A brief summary of the purchase intention changes on GM foods is presented in. About 32.46% of consumers changed their purchase intention. Specially, about 7.37%, 3.92%, and 3.80% of the consumers changed from positive to negative, that is, from “purchase” to “unwilling,” from “unclear” to “unwilling,” and from “willing” to “unclear,” respectively. About 5.83%, 3.57%, and 7.97% of consumers changed from negative to positive, that is, from “not purchase” to “unclear,” from “unclear” to “willing,” and from “not purchase” to “willing,” respectively.

Change in intention to purchase GM soybean oil.

Notes: a/b: a indicates the number of respondents. b indicates the corresponding proportion.

The differences of consumers’ purchase intention among three groups change significantly after receiving the information. The proportions of initial purchase behavior and purchase intention of the three groups are presented in Table 3. There are no significant differences for the proportion of “yes” and “no” in the initial purchase behavior among three groups. However, compared with subsample1, the proportions of “willing to purchase” in subsample2 and subsample3 are lower significantly after receiving the information, while the proportions of “unwilling to purchase” are higher significantly. Additionally, compared with subsample2, the proportion of “unclear” in the initial purchase behavior are higher significantly in both subsampble1 and subsample3. But there are no significant differences for the proportion of “unclear” among three groups after receiving the information. Therefore, the results show that the provided information about benefits and risks of GM foods may affect consumers intention to purchase GM soybean oil.

**Table 3.** Proportion of respondents with various purchase intention and test of difference in ration between two samples

	Proportion of respondents with various purchase intention (%)				Test of difference in ratio between two subsamples		
	Total sample (N = 841)	Subsample1^a^ (N = 413)	Subsample2 ^b^ (N = 213)	Subsample3 ^c^ (N = 215)	①-②	①-③	②-③
		①	②	③			
Initial purchase behavior							
1 = yes	0.319	0.325	0.347	0.279	−0.022	0.046	0.068
2 = no	0.565	0.555	0.582	0.567	−0.027	−0.012	0.015
3 = unclear	0.117	0.121	0.070	0.154	0.051**	−0.033	−0.084***
Purchase intention, after receiving the information							
1 = willing	0.322	0.380	0.282	0.251	0.098**	0.129***	0.031
2 = unwilling	0.540	0.475	0.582	0.623	−0.107***	−0.148***	−0.041
3 = unclear	0.138	0.145	0.136	0.126	0.009	0.019	0.01

**p < 0.05, ***p < 0.01. ^a^ Respondents receive information from *GM department or experts*. ^b^ Respondents receive information from *environmental organizations*. ^c^ Respondents receive information from n*on-GM individuals.*

Regarding information consistency, the consumers who reported completely inconsistent, inconsistent, neutral or unclear, consistent, and completely consistent account for 4.64%, 19.02%, 38.64%, 35.32%, and 2.38%, respectively (see Table 2). In terms of source credibility, given the probability of participants (49.1%, 25.3%, and 25.6%), about 21.6% participants received information about benefits of GM foods from GM department or experts, and trust in this type of source. About 13.3% participants received information about risks of GM foods from environmental organizations, and trust in the source. While only 5% participants received information about risks of GM foods from Non-GM individuals, and trust in this type of source.

### 4.2. Estimation Results and Discussion

Using the maximum likelihood estimation, the multinomial logit model was conducted, and the marginal effects were calculated. The mean and standard deviation of all variables see Table 1, which mean the values of each variable in the given sample are not all the same. The VIF value is 2.01 and the condition number is 19.6, which mean there is no multicollinearity among the explanatory variables. Likelihood ratio (LR) test is for comparing to the model without any independent variables. The Chi-squared test (*p* < .000) showed that the multinomial logistic model is useful in analyzing the change in consumer purchase intention. The results of the multinomial logistic regression with robust standard errors are presented in Table 4.

**Table 4.** The estimation results of purchase intention change using multinomial logistic regression

Variable	Estimates		Marginal effects		
	Positive change	Negative change	No change	Positive change	Negative change
Information consistency					
*Completely inconsistent*	−0.083	0.463	−0.037	−0.021	0.059
	(0.567)	(0.456)	(0.082)	(0.074)	(0.056)
*Inconsistent*	0.551**	0.323	−0.093**	0.065*	0.027
	(0.269)	(0.298)	(0.046)	(0.035)	(0.036)
*Neutral*	0.301	0.085	−0.041	0.038	0.004
	(0.232)	(0.246)	(0.039)	(0.030)	(0.030)
*Completely consistent*	−0.539	−0.339	0.093	−0.063	−0.030
	(0.811)	(0.788)	(0.118)	(0.108)	(0.098)
Source					
*GM department or experts*	0.245	−0.033	−0.023	0.033	−0.009
	(0.299)	(0.276)	(0.047)	(0.039)	(0.033)
*Environmental organizations*	−0.174	−0.220	0.041	−0.018	−0.023
	(0.375)	(0.342)	(0.058)	(0.048)	(0.041)
*Source credibility*	−0.565	−0.537	0.116	−0.062	−0.053
	(0.614)	(0.526)	(0.090)	(0.080)	(0.064)
*Credibility of GM department or experts*	1.090	−0.105	−0.108	0.146*	−0.037
	(0.675)	(0.611)	(0.102)	(0.088)	(0.074)
*Credibility of environmental organizations*	0.612	0.491	−0.116	0.070	0.047
	(0.743)	(0.640)	(0.111)	(0.097)	(0.078)
*Male*	−0.270	0.113	0.018	−0.038	0.020
	(0.201)	(0.203)	(0.032)	(0.026)	(0.024)
*Age*	−0.029**	−0.015	0.005**	−0.003*	−0.001
	(0.014)	(0.014)	(0.002)	(0.002)	(0.002)
*Professional college or above*	0.436	0.286	−0.076*	0.051	0.025
	(0.295)	(0.271)	(0.046)	(0.038)	(0.033)
*Low-income group*	0.221	−0.112	−0.013	0.032	−0.019
	(0.399)	(0.468)	(0.070)	(0.051)	(0.056)
*Lower-middle group*	0.125	0.758**	−0.09	0.000	0.09**
	(0.342)	(0.357)	(0.058)	(0.044)	(0.042)
*Middle-income group*	−0.030	−0.348	0.038	0.004	−0.042
	(0.284)	(0.329)	(0.049)	(0.037)	(0.040)
*High-income group*	−0.456*	0.114	0.038	−0.063*	0.024
	(0.248)	(0.257)	(0.040)	(0.032)	(0.031)
*Child*	−0.202	−0.136	0.036	−0.024	−0.012
	(0.204)	(0.207)	(0.033)	(0.026)	(0.025)
*Main edible oil*	−0.123	0.522**	−0.039	−0.028	0.067**
	(0.196)	(0.221)	(0.033)	(0.025)	(0.026)
*Southern region*	0.093	−0.085	−0.002	0.014	−0.012
	(0.220)	(0.230)	(0.036)	(0.028)	(0.028)
*Central region*	0.022	−0.157	0.013	0.006	−0.020
	(0.308)	(0.324)	(0.051)	(0.040)	(0.039)
Constant	−0.978	−1.476**			
	(0.647)	(0.661)			
Observations	841				
LR chi2	89.42***				

The base outcome is no change. Robust Standard errors in parentheses. *p < 0.10, **p < 0.05, ***p < 0.01.As for dummy variables, the marginal effects are for discrete change from 0 to 1.

Regarding information consistency, the marginal effect of *Inconsistent* is negative for “no change” group and positive for “positive change” group, significantly. While the marginal effect of *Completely inconsistent, Neutral, Completely consistent* are not significant. Our results show that systematic purchase intention changes occurred in response to the inconsistent information rather than completely inconsistent information, neutral information and completely consistent information. The results are in alignment with the theoretical analysis of Cognitive Response Theory^10^ and Elaboration Likelihood Model,^11^ which indicate that prior knowledge is an important factor affecting information processing of consumers. When the difference between the persuasion information and the initial attitude of the persuasion object is moderate, the attitude change is more susceptible to the information. The results are also similar with findings of Priester et al.,^12^ who thinks that external information leads to individual comparing it with the original memory based on the initial information. But we haven’t found other similar results yet.

In terms of source credibility, the marginal effect of *Credibility of GM department or experts* is 0.146 and significant for “positive change” group. The results show that it is easier to promote consumers to change their intention to purchase GM soybean oil in a positive direction that trust in GM department or experts releasing information about benefits of GM foods. While trusting in those sources can hinder consumer to change attitude in a negative direction. So far, related studies did not find a consistent effect of information on attitude change.^16^ Some studies found no systematic attitude changes in response to the information with the same or different persuasiveness power, and even to different types of information.^33,34,37^ Miles, et al.,^38^ Wuepper, et al.^39^ also found no effect of attitude change was observed by providing information about the traceability of GM ingredients or GM function. But Qin and Brown^40^ obtained a small attitude change effect in response to information material about the specific application of GM salmon. Zheng, et al.^21^ indicated that the media coverage with the “event of Golden rice in 2012” as an example helped consumers shape their negative perceptions toward GM foods. Though there has been no clear consensus on its effect, some scholars indicated the positive information helps form a positive attitude because of a reduction in risk perceptions and an increase in benefit perceptions.^45,57,58^ On the other hand, with limited knowledge of GM foods, the consumers may have to rely on the credibility of certain information sources to obtain information before making a purchase decision.^13,14,41^ The information from experts may affect consumers’ comprehension of scientific information, and lead to a positive effect on attitude change.^43,46^ This finding can also be confirmed by the theoretical analysis of Hovland’s Theories of Attitude Change.^8^

Additionally, the marginal effect of *Professional college or above* is −0.076 and significant for “no change” group, while the marginal effect of *Age* is 0.005 and −0.003 significantly for “no change” group and “positive change” group. The results indicate that well-educated respondents and elders prefer to keep initial attitude, while youngster are easier to change their purchase intention of GM soybean oil in the positive direction. The marginal effect of *Lower-middle group*is 0.09 and significant for “negative change” group, while the marginal effect of *High-income group* is −0.063 significantly for “positive change” group. The results show that compared with respondents with higher-middle income, respondents with lower-middle income are easier to change their purchase intention in the negative direction, while respondents with higher income are more reluctant to change in the positive direction. In our survey, we find purchasing organic foods has significant positive correlation with high-income, so those respondents with high-income would consume non-GM oil such as organic foods instead of GM soybean oil. The marginal effect of *Main edible oil* is 0.067 and significant for “negative change” group, which means the respondents with soybean oil being the main edible oil are more willing to change their purchase intention in a negative direction. Currently, it has not yet reached a consensus on how demographic characteristics affect consumer attitudes toward GMFs.^31,41,59,60^

### 4.3. Heterogeneity Check: By *Group according to the Consumers’ Initial Purchase Behavior*

Initial purchase behavior is a key factor to consumers’ attitude change. This section discusses the heterogeneity of marginal effect by group according to the consumers’ initial purchase behavior on GM soybean oil. The heterogeneity results are presented in Table 5.

**Table 5.** Heterogeneity check: marginal effects of subsample with different initial purchase intention

Variable	Initial purchase behavior is “yes”	Initial purchase behavior is “no”	Initial purchase behavior is “unclear”		
	Negative change (base = no change)	Positive change (base = no change)	No change	Positive change	Negative change
*Completely inconsistent*	0.157	−0.053	−0.422***	0.215	0.208
	(0.142)	(0.100)	(0.066)	(0.342)	(0.350)
*Inconsistent*	0.217**	0.045	0.054	−0.030	−0.024
	(0.108)	(0.061)	(0.188)	(0.179)	(0.164)
*Neutral*	0.142*	−0.003	0.157	0.175	−0.332**
	(0.081)	(0.048)	(0.140)	(0.129)	(0.129)
*Completely consistent*	0.078	0.012			
	(0.211)	(0.126)			
*GM department or experts*	0.108	0.044	0.031	0.279*	−0.310**
	(0.101)	(0.061)	(0.153)	(0.155)	(0.143)
*Environmental organizations*	0.048	−0.061	−0.044	0.328	−0.283**
	(0.118)	(0.068)	(0.184)	(0.218)	(0.133)
*Source credibility*	−0.284*	−0.206	−0.284	0.332	−0.049
	(0.160)	(0.139)	(0.198)	(0.215)	(0.177)
*Credibility of GM department or experts*	−0.004	0.476**	0.363	−0.143	−0.220
	(0.193)	(0.229)	(0.330)	(0.254)	(0.175)
*Credibility of environmental organizations*	0.247	0.361	0.207	−0.063	−0.144
	(0.219)	(0.263)	(0.407)	(0.348)	(0.272)
Socio-demographic variables	Yes	Yes	Yes		
Other control variables	Yes	Yes	Yes		
Observations	268	475	98		
Model	logistic	logistic	Multinomial logistic		
LR chi2	38.67***	52.24***	1035.30***		

The base outcome is no change. Robust Standard errors in parentheses. *p < 0.10, **p < 0.05, ***p < 0.01.

For the consumers whose initial purchase behavior is “yes,” compared with consistent information, inconsistent information and neutral information are easier to promote consumers to change their purchase intention on GM soybean oil in a negative direction. For the consumers who are unclear about their initial purchase behavior, compared with consistent information, completely inconsistent information is easier to change their intention to purchase GM soybean oil, while neutral information is easier to change their purchase intention in a negative direction. Finally, for the consumers whose initial purchase behavior is “no,” it is easier to promote them to change their purchase intention of GM soybean oil in a positive direction that trust in GM department or experts releasing information about benefits of GM foods.

### 4.4. Heterogeneity Check: By *Group according to the Information Cards Provided to Consumers*

The results of descriptive statistics show that consumers’ purchase intention on GM soybean oil change systematically after receiving information about benefits and risks of GM foods. In order to test whether the purchase intention change is affected by heterogeneity information, this section discusses the heterogeneity of marginal effect by group according to the information cards provided to consumers. Heterogeneity results are presented in Table 6.

**Table 6.** Heterogeneity check: marginal effects of subsample with respondents receiving information from different type of sources

Variable	Subsample1 with respondents receiving information from *GM department or experts*			Subsample2 with respondents receiving information from *environmental organizations*			Subsample3 with respondents receiving information from n*on-GM individuals*		
	No change	Positive change	Negative change	No change	Positive change	Negative change	No change	Positive change	Negative change
*Completely inconsistent*	−0.042	−0.157**	0.198	0.013	−0.022***	0.008	−0.249	0.257	−0.008
	(0.148)	(0.065)	(0.150)	(0.045)	(0.006)	(0.045)	(0.155)	(0.163)	(0.100)
*Inconsistent*	−0.066	−0.003	0.069	−0.007	0.018	−0.011	−0.184*	0.187**	−0.002
	(0.072)	(0.055)	(0.062)	(0.029)	(0.015)	(0.024)	(0.100)	(0.095)	(0.057)
*Neutral*	−0.019	−0.029	0.048	−0.021	0.012	0.008	0.009	0.045	−0.054
	(0.061)	(0.049)	(0.046)	(0.022)	(0.008)	(0.020)	(0.060)	(0.043)	(0.046)
*Completely consistent*	−0.028	−0.034	0.063	−0.023	−0.016***	0.039	0.240***	−0.072***	−0.168***
	(0.186)	(0.137)	(0.178)	(0.077)	(0.004)	(0.077)	(0.033)	(0.022)	(0.028)
*Source credibility*	−0.009	0.081*	−0.072**	0.001	0.002	−0.002	0.063	−0.020	−0.043
	(0.053)	(0.046)	(0.035)	(0.019)	(0.005)	(0.018)	(0.058)	(0.030)	(0.050)
Socio-demographic variables	Yes	Yes	Yes	Yes	Yes	Yes	Yes	Yes	Yes
Other control variables	Yes	Yes	Yes	Yes	Yes	Yes	Yes	Yes	Yes
Observations	413			213			215		
Wald chi2	51.09**			4789.76***			1097.86***		

Robust Standard errors in parentheses. *p < 0.10, **p < 0.05, ***p< 0.01.

For the consumers only receiving card A with information about benefits of GM foods from GM department or experts, compared with consistent information, completely inconsistent information can hinder consumer to change attitude in a positive direction. While, it is easier to promote consumers to change their intention to purchase GM soybean oil in a positive direction that trust in GM department or experts. For the consumers only receiving card B with information about risks of GM foods from environmental organizations, compared with consistent information, both completely inconsistent information and completely consistent information can hinder consumer to change attitude in a positive direction. For the consumers only receiving card C with information about risks of GM foods from by non-GM individuals, compared with consistent information, the attitude changes are more susceptible to the inconsistent information, which is easier to promote consumers change attitude in a positive direction. While, completely consistent information can hinder consumers to change attitude.

## 5. Conclusions and Implications

This study discusses how Chinese consumers change his/her intention to purchase GM soybean oil after receiving information communication about risks or benefits of GM foods. An empirical analysis is conducted using an eastern coastal province in China, Jiangsu. In general, after receiving new information, there are significant structural changes in purchase intention of GM soybean oil. About 17.36% consumers change in positive direction, and about 15.10% consumers change in negative direction. Our results showed that systematic purchase intention changes occurred in response to the inconsistent information rather than completely inconsistent information, neutral information, and completely consistent information. Consumer’s ability to process information is affected by the prior knowledge. When the difference between the persuasion information and the initial attitude is moderate, the attitude change is more susceptible to the new information. Furthermore, trust in biotechnology research institutes, government departments about GM, and GM experts are easier to promote consumers to change their purchase intention of GM soybean oil in a positive direction.

To contribute to this literature, this study provides scientific insights by investigating how consumers’ purchase intention of GM soybean oil response to information about risks and benefits of GM foods, and reveals the influence of information consistency and source credibility on consumer intention to purchase GM foods. The results signify the importance of external information on consumers’ attitude change. Thus, the theoretical framework and empirical analysis can be referred to for other countries or areas to analyze the effect of information on consumers’ change in purchase intention of GM foods. Additionally, agency leaders may be pleased to know that the information about benefits of GM foods from GM department or experts can lead to consumer attitude change in a positive direction. Because taking into account of social acceptance is critical for agencies to increase investment in R&D of transgenesis. Some insights can be helpful for policy design. To avoid spreading rumors and misleading consumers, the regulation and supervision of information sources should be further strengthened. Specifically, entities that fabricate information and data, spread rumors, or maliciously twist research results should be seriously punished through economic, administrative, and legal penalties. Additionally, agencies should include public-involved policies to gain their support, and encourage the public to participate in the supervision of information sources. A dedicated webpage on Authoritative Attention to GM can be opened, where the public can report rumors and misleading information any time and the new research findings and research trends of transgenesis can be released. Regarding some GM events, the reports should be available to the public, in particular, the causes and possible actions should be delivered to the public by means of TV column and online networks such as Blog, Microblog, WeChat, etc. Information about and outcomes of transgenic workshops can be released by online videos, which can reach more people and be available for a long time.

There are opportunities for some future research. This paper is restricted to consumer intention to purchase GM soybean oil. Relevant research may expand this approach to other GM foods.
